# Reduced NRF2 expression suppresses endothelial progenitor cell function and induces senescence during aging

**DOI:** 10.18632/aging.102234

**Published:** 2019-09-08

**Authors:** Ruiyun Wang, Lihua Liu, Hongxia Liu, Kefei Wu, Yun Liu, Lijuan Bai, Qian Wang, Benming Qi, Benling Qi, Lei Zhang

**Affiliations:** 1 Department of Geriatrics, Union Hospital, Tongji Medical College, Huazhong University of Science and Technology, Wuhan 430022, China; 2 Department of Otorhinolaryngology, First People’s Hospital of Yunnan Province, Kunming, Yunnan 650000, China; 3 Division of Gastroenterology, Union Hospital, Tongji Medical College, Huazhong University of Science and Technology, Wuhan 430022, China

**Keywords:** endothelial progenitor cells, aging, oxidative stress, NRF2, NLRP3 inflammasome

## Abstract

Aging is associated with an increased risk of cardiovascular disease. Numerical and functional declines in endothelial progenitor cells (EPCs) limit their capacity for endothelial repair and promote the development of cardiovascular disease. We explored the effects of nuclear factor (erythroid-derived 2)-like 2 (NRF2) on EPC activity during aging. Both *in vitro* and *in vivo*, the biological functioning of EPCs decreased with aging. The expression of NRF2 and its target genes (*Ho-1*, *Nqo-1* and *Trx*) also declined with aging, while Nod-like receptor protein 3 (NLRP3) expression increased. Aging was associated with oxidative stress, as evidenced by increased reactive oxygen species and malondialdehyde levels and reduced superoxide dismutase activity. *Nrf2* silencing impaired the functioning of EPCs and induced oxidative stress in EPCs from young mice. On the other hand, NRF2 activation in EPCs from aged mice protected these cells against oxidative stress, ameliorated their biological dysfunction and downregulated the NLRP3 inflammasome. These findings suggest NRF2 can prevent the functional damage of EPCs and downregulate the NLRP3 inflammasome through NF-κB signaling.

## INTRODUCTION

With the increased longevity of humans, the percentage of people entering the 65-and-older age group is increasing rapidly, and this trend is expected to continue for several decades. Cardiovascular disease (CVD) remains the leading cause of death in the elderly, and treatment is costly [1]. Aging is the most important independent risk factor for CVD, due to its remarkable effects on the heart and arterial system. Aging cardiovascular tissues undergo a series of pathological changes, including hypertrophy, altered left ventricular diastolic function, a diminished left ventricular systolic reverse capacity, increased arterial stiffness and impaired endothelial function [1–3]. The reduced endothelial function with aging contributes to the development of CVD, so maintaining the normal endothelial integrity is an important therapeutic approach to reduce the age-related risk of CVD.

Endothelial progenitor cells (EPCs) are thought to promote postnatal neovascularization and maintain endothelial integrity and function. These cells have aroused the interest of researchers, especially given the limited regenerative capacity of mature endothelial cells. It has been suggested that EPCs not only foster the continuous recovery of the endothelium after injury/damage, but also stimulate angiogenesis [4].

The function and number of circulating EPCs decreases with aging [5–8]. Aging impairs the ability of EPCs to regenerate and migrate to damaged blood vessels and ischemic areas to repair the vasculature and promote angiogenesis [9]. Aging EPCs exhibit reduced capacities for colony formation, migration, adhesion, reendothelialization and incorporation into tube-like structures [6, 7]. Therefore, therapeutic interventions that stimulate EPCs to enhance endothelial repair in elderly individuals have important clinical implications for the aging population. Different mechanisms of EPC senescence have been reported, including telomerase shortening, age-related declines in pro-angiogenic factors, increased oxidative stress, reduced nitric oxide (NO) bioavailability and chronic low-grade inflammation [9]. However, the complex molecular network responsible for EPC senescence requires further investigation.

Nuclear factor erythroid 2-related factor 2 (NRF2) is a transcription factor that can be activated by cellular stressors such as oxidative stimuli [10, 11], and regulates the basal and inducible expression of an array of antioxidant and detoxification enzymes. NRF2 is regulated by a cytosolic repressor protein called Kelch-like ECH-associated protein 1 (KEAP1), which functions as a primary redox sensor. In combination with other adaptor components, KEAP1 promotes the ubiquitination and proteasomal degradation of NRF2 under basal conditions [12]. NRF2 has been widely studied for its anti-aging effects and its ability to alleviate age-related diseases [13, 14]. Recent studies have indicated that NRF2 can increase the lifespan by protecting against oxidative stress [15, 16]. Increased NRF2 activity in an AKT/GSK-3β/FYN pathway was thought to be responsible for the enhanced therapeutic effects of CXCR7-transduced EPCs in diabetic limb ischemia [17]. However, whether and how NRF2 regulates EPC function during aging remains to be studied.

Here, we monitored NRF2 expression and EPC functioning during aging. We then investigated the effects of altering NRF2 expression on the migration, proliferation, secretion, tube-forming capacity and intracellular signaling of young and aged EPCs.

## RESULTS

### Characteristics of early EPCs

Murine mononuclear cells were isolated and cultured for seven days, after which they resembled cobblestones (Supplementary Figure 1A). To confirm that the adherent cells were EPCs, we performed dual staining for 1,1′-dioctadecyl-3,3,3′,3′-tetramethylindo-carbocyanine-labeled acetylated low-density lipoprotein (Dil-acLDL) and lectin. Most of the cells cultured for seven days in EGM-2 medium were double-positive for Dil-acLDL uptake and lectin binding affinity (Supplementary Figure 1B).

### Characterization of EPC survival, migration, proliferation, secretion and angiogenesis revealed age-related vulnerability

**Figure 1:**
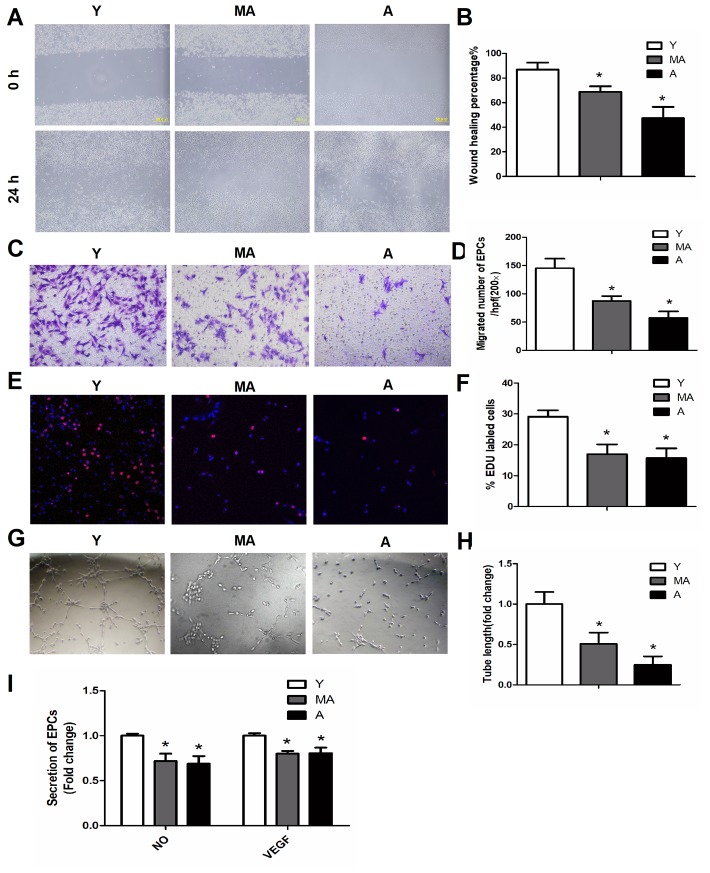
**The migration, proliferation, secretion and angiogenesis of EPCs declined with aging.** (**A**, **B**) Scratch test of EPCs isolated from young, middle-aged and aged mice, at 0 and 24 h. Magnification, ×100. (**C**, **D**) Representative images of crystal violet-stained migrated cells, as assessed by a Transwell assay. Magnification, ×400. The number of migrated EPCs was calculated. (**E**, **F**) The proliferative ability of EPCs was assessed based on the percentage of EdU-positive cells (red). Scale bar: 100 μm. (**G**, **H**) Representative images displaying tube-forming activity. Magnification, ×100. (**I**) The secretion of VEGF and NO into the supernatants of cultured EPCs. *P<0.05. n=5. Y, young; MA, middle-aged; A, aged.

Bone marrow EPCs were isolated from three groups of mice: 3- to 4-month-old (young), 12- to 14-month-old (middle-aged) and 21- to 24-month-old (aged) mice. The EPC properties of migration, proliferation, secretion and angiogenesis were then investigated *in vitro*. Both a scratch healing assay and a Transwell assay revealed a progressive decline in EPC migration with aging (Figure 1A–1D). A 5-ethynyl-2′-deoxyuridine (EdU) assay demonstrated that the proliferation of EPCs also declined during aging (Figure 1E, 1F). Further, the tube-forming ability or angiogenesis potential of EPCs diminished with age (Figure 1G, 1H). Accordingly, the secretion of nitric oxide (NO) and vascular endothelial growth factor (VEGF) by EPCs decreased with increasing age (Figure 1I). These data indicated that the migration, proliferation, angiogenesis and secretion functions of EPCs all decline during aging.

### The age-related decline in EPC properties correlated with reduced NRF2 expression *in vitro*

**Figure 2:**
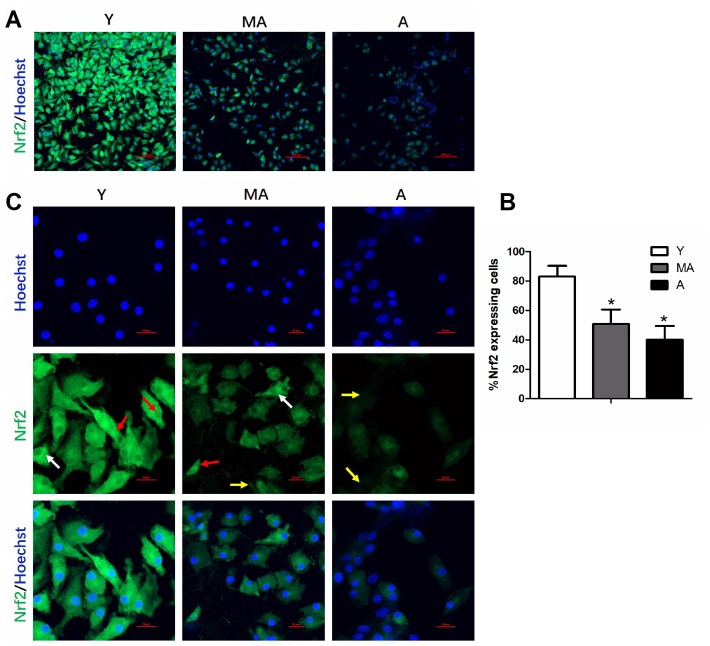
**NRF2 expression in cultured EPCs was reduced with advancing age.** Low-magnification images of NRF2 expression in different age groups are shown in (**A**), with quantification in (**B**). High-magnification confocal images of NRF2 expression are shown in (**C**). NRF2 expression was detected in the cytoplasm (white arrows) and/or nuclei (red arrows) in younger cells, while little or no NRF2 expression was detected in middle-aged and aged cells (yellow arrows). *P<0.05. n=5. Y, young; MA, middle-aged; A, aged. Scale Bar: (**A**) 100 μm, (**C**) 20 μm.

We performed immunocytochemistry to examine NRF2 expression in the three age groups of EPCs (Figure 2). Qualitatively, NRF2 expression decreased with age in EPCs (Figure 2A). A quantitative analysis confirmed the age-related reduction in the percentage of cultured EPCs expressing NRF2 (Figure 2B). These results closely mirrored the decreases in EPC survival and proliferation with age. Confocal imaging revealed high NRF2 levels in the cytoplasm and/or nuclei of young cells, whereas little or no NRF2 expression was observed in middle-aged and aged cells (Figure 2C). Western blotting and real-time PCR also demonstrated that the protein and mRNA levels of NRF2 in EPCs declined with age, as shown in Figure 6E–6G.

### Aging impaired angiogenesis in response to ischemia

**Figure 3:**
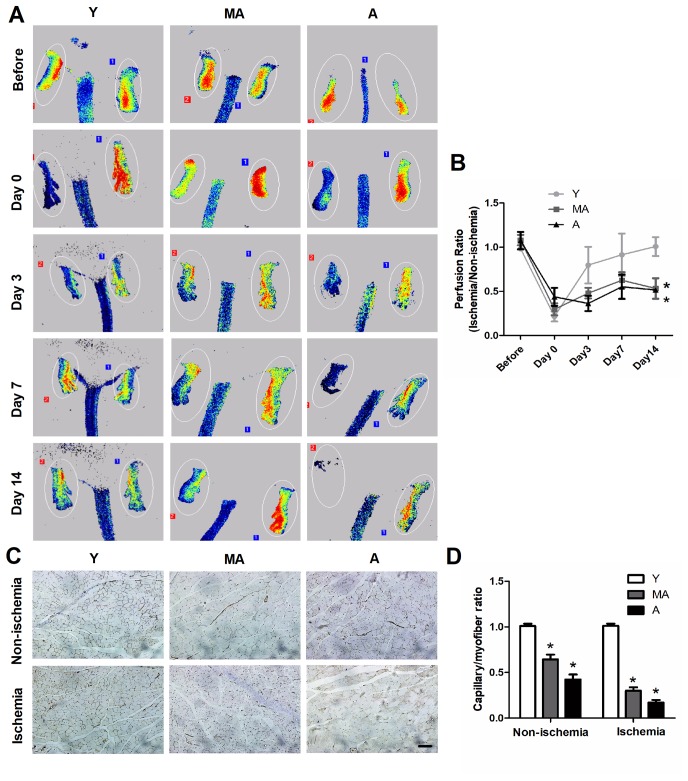
***In vivo* characterization of EPC angiogenesis.** (**A**) Serial laser speckle blood flow imaging revealed that the ischemic hindlimbs of aged mice exhibited lower perfusion signals than those of younger mice. (**B**) The ratio of ischemic to normal blood flow decreased with aging. (**C**) On postoperative day 14, immunostaining was conducted to evaluate the capillaries in non-ischemic and ischemic thigh adductor muscles. (**D**) Quantitative analyses revealed that aging reduced the capillary density in both non-ischemic and ischemic muscles. *P<0.05. n=5. Y, young; MA, middle-aged; A, aged. Scale Bar: (**C**) 100 μm.

We used a mouse hindlimb ischemia model to evaluate the impact of aging on ischemia-induced vascular regeneration. Serial laser speckle blood flow imaging analyses demonstrated that the recovery of the ischemic/non-ischemic blood flow ratio in aged mice remained impaired throughout the follow-up period (Figure 3A, 3B). On postoperative day 14, quantitative immunostaining revealed that the capillary density in both non-ischemic and ischemic muscles was lower in aged mice than in young mice (Figure 3C, 3D), suggesting that aging impairs vascular regeneration.

### Alteration of *Nrf2* expression significantly impacted EPC survival and function

Given the significant declines in NRF2 expression in aging EPCs, we further assessed the function of NRF2 through *in vitro* knockdown (RNA interference) and overexpression assays.

### Downregulation of Nrf2 induced senescence in young EPCs

**Figure 4:**
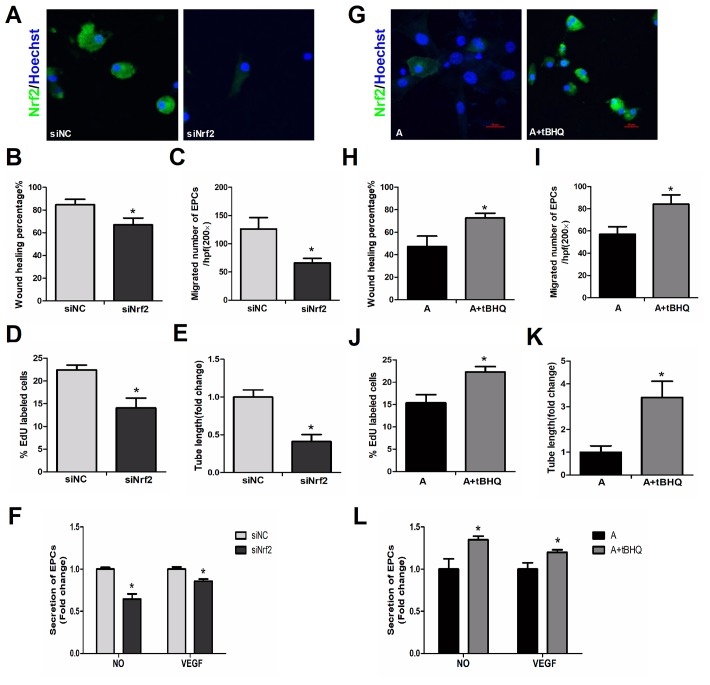
**Effects of altered NRF2 expression on EPC survival and function.** (**A**) Nrf2 expression with or without knockdown of *Nrf2* in young cells. EPC function was evaluated by a scratch wound-healing assay (**B**), Transwell assay (**C**), EdU assay (**D**), tube formation assay (**E**) and secretion assay (**F**) in young cells treated with negative control siRNA (siNC) and *Nrf2* siRNA (siNrf2). (**G**) NRF2 expression with and without NRF2 overexpression in aged cells, with parallel results from a scratch wound-healing assay (**H**), Transwell assay (**I**), EdU assay (**J**), tube formation assay (**K**) and secretion assay (**L**) conducted in aged cells that were untreated or treated with tBHQ. *P<0.05. Y, young; MA, middle-aged; A, aged. A+tBHQ, aged EPCs treated with tBHQ. Scale bar: 20 μm.

Targeted small interfering RNA (siRNA) was used to silence *Nrf2* in young EPCs. Figure 4A display NRF2 expression with and without the knockdown. The *Nrf2* knockdown rendered young cells similar to aged cells, with lower capacities for migration (Figure 4B, 4C), proliferation (Figure 4D), angiogenesis (Figure 4E) and NO and VEGF secretion (Figure 4F) than negative silencing controls.

### Upregulation of NRF2 restored the function of aged EPCs

Tert-butylhydroquinone (tBHQ, MedChemExpress, Shanghai, China) is one of the most studied NRF2 inducers. This compound exists in the body and is also widely used as a food preservative. When tBHQ was used to upregulate NRF2 in aged EPCs (Figure 4G), the migration (Figure 4H, 4I), proliferation (Figure 4J), angiogenesis (Figure 4K) and secretion (Figure 4L) of these cells significantly improved, making them akin to younger cells. These data indicated that simply altering intrinsic NRF2 expression could significantly change EPC survival and function, demonstrating the importance of NRF2 in maintaining EPC activity during aging.

### NRF2 suppressed oxidative stress in EPCs during aging

**Figure 5:**
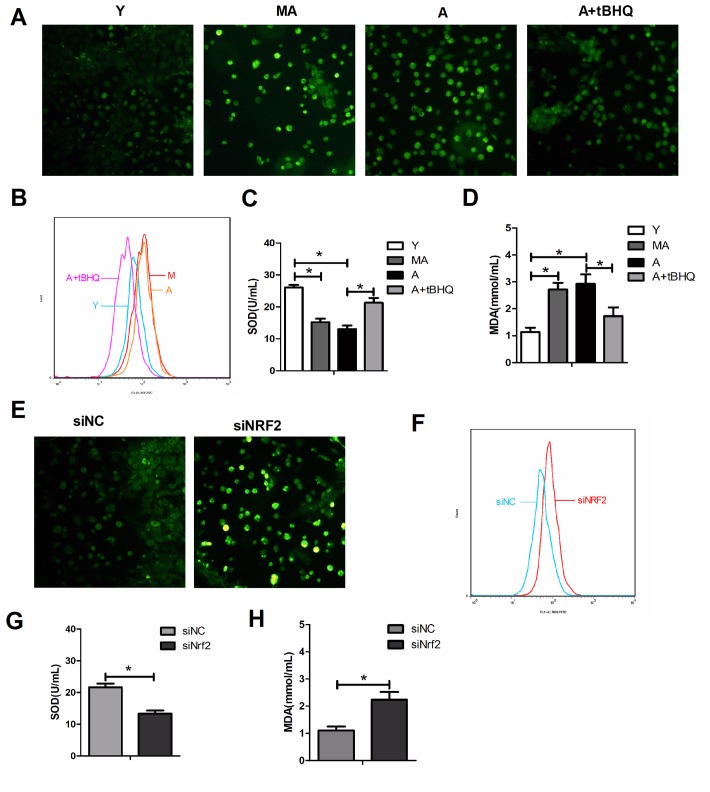
**NRF2 protected against oxidative stress in EPCs during aging.** ROS levels were detected by fluorescence imaging (green, 100×) (**A**, **E**) and flow cytometry (**B**, **F**) in different groups of EPCs. The levels of SOD (**C**, **G**) and malondialdehyde (**D**, **H**) were also assessed in the supernatants of EPCs. *P<0.05. Y, young; MA, middle-aged; A, aged. A+tBHQ, aged EPCs treated with tBHQ.

We then examined the levels of oxidative stress in EPCs from mice of different age groups. EPCs from the middle-aged and aged groups had greater reactive oxygen species (ROS) and malondialdehyde levels and lower superoxide dismutase (SOD) levels than EPCs from the young group (Figure 5A–5D). When aged EPCs were treated with tBHQ, NRF2 expression increased, ROS and malondialdehyde levels decreased and SOD levels increased (Figure 5A–5D). On the other hand, after the silencing of *Nrf2* in young EPCs, ROS and malondialdehyde levels increased and SOD levels decreased (Figure 5E–5H). These observations suggested that NRF2 prevents oxidative stress in EPCs during aging.

### NRF2 suppressed the NLRP3/NF-κB pathway

**Figure 6:**
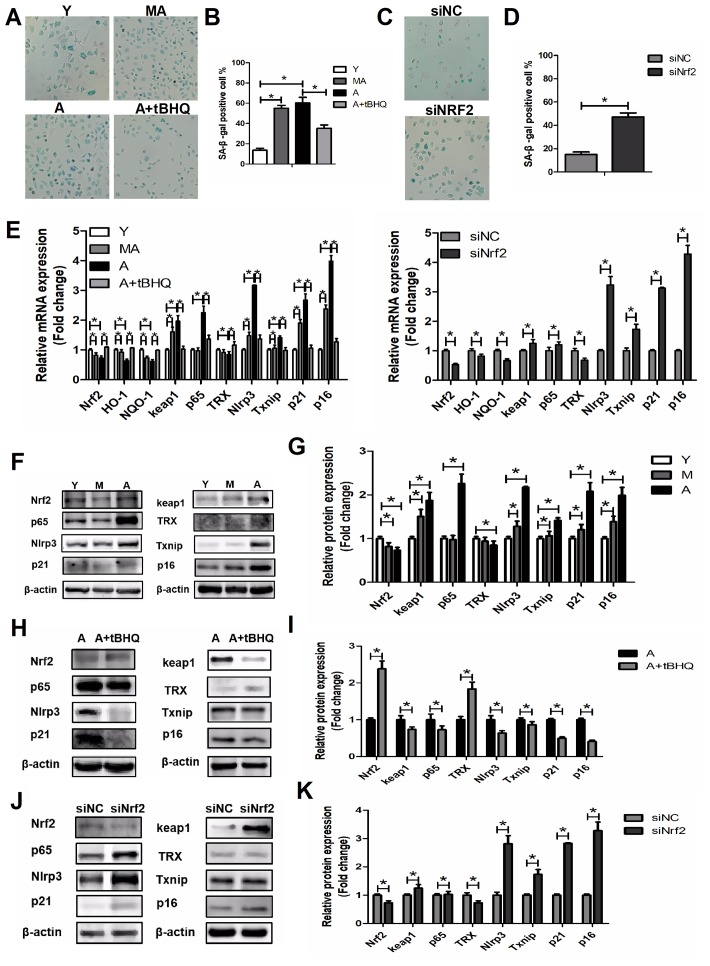
**NRF2 suppressed EPC senescence.** (**A**–**D**) β-galactosidase staining of Y, MA, A, A+tBHQ, siNC and siNrf2 EPCs. (**E**) Expression of *Nrf2*, *Ho-1*, *Nqo-1*, *Keap1*, *p65*, *Trx*, *Nlrp3*, *Txnip*, *p21* and *p16* mRNA in Y, MA, A, A+tBHQ, siNC and siNrf2 EPCs. (**F**–**K**) Representative Western blots and quantitative analysis of NRF2, KEAP1, p65, TRX, NLRP3, TXNIP, p21 and p16 levels in Y, MA, A, A+tBHQ, siNC and siNrf2 EPCs. *P<0.05. Y, young; MA, middle-aged; A, aged. A+tBHQ, aged EPCs treated with tBHQ.

We observed that the activation of NRF2 alleviated the symptoms of aging in EPCs by restoring the biological functions of migration, proliferation, secretion and angiogenesis, while silencing *Nrf2* appeared to induce senescence in young EPCs. To confirm that NRF2 inhibits cellular senescence, we assessed several biomarkers of senescence after stimulating or silencing NRF2 expression. Upregulating NRF2 in aged EPCs prevented cellular senescence, as evidenced by the levels of senescence-associated β-galactosidase and the mRNA and protein levels of p16 and p21 (Figure 6A, 6B, 6E–6G). In contrast, silencing *Nrf2* in young EPCs increased cellular senescence (Figure 6C–6E, 6H, 6I).

To evaluate the molecular mechanisms whereby NRF2 prevents cellular senescence, we investigated NLRP3, p65, TXNIP and TRX levels in young, middle-aged and aged EPCs. NLRP3, p65 and TXNIP levels were higher and TRX levels were lower in middle-aged and aged EPCs than in young EPCs (Figure 6E–6G). We found that tBHQ reduced NLRP3, p65 and TXNIP and increased TRX expression in aged EPCs (Figure 6E, 6H, 6I), while silencing *Nrf2* in young EPCs upregulated NLRP3, p65 and TXNIP and downregulated TRX expression (Figure 6E, 6J, 6K). From these results, we concluded that activating NRF2 could inhibit aging by reducing the expression of the NLRP3 inflammasome via the NF-κB pathway, while silencing *Nrf2* could increase NLRP3 expression, causing senescence.

## DISCUSSION

In this study, we found that age-related loss of NRF2 expression reduced the functioning of EPCs and thereby impaired angiogenesis. RNA interference data indicated that downregulating *Nrf2* in young EPCs impaired their migration, proliferation, secretion and tube formation. Moreover, increasing NRF2 expression in aged EPCs downregulated NLRP3 and was sufficient to prevent the age-related dysfunction of these cells. These results, for the first time, identify NRF2 as an important intrinsic factor contributing to the decline in EPC function during aging. Our findings not only shed light on fundamental aspects of EPC biology, but also have important implications for efforts to improve EPC function with advancing age.

Previous studies have shown that older individuals have significantly lower concentrations of CD133^+^/VEGFR2^+^ cells, CD31^+^/c-Kit^+^ cells and CD34^+^/VEGFR2^+^ cells in peripheral blood or bone marrow, and that the colony-forming and cellular migratory capacities of EPCs decline with age in humans [6, 7, 18, 19]. We characterized how the EPC population changes across the lifespan in multiple groups of animals at various stages of aging. By systematically examining EPC survival, proliferation, migration, secretion and angiogenesis *in vitro* and *in vivo*, we demonstrated that EPC function declines considerably with age. Critically, we found that the specific temporal pattern of EPC deterioration was associated with impaired neovascularization in response to ischemia in aging mice.

Our results indicated that the decline in EPC function with aging correlated with reduced expression of NRF2 and its target genes (*Ho-1* and *Nqo-1*) and increased levels of ROS in EPCs. Inhibiting *Nrf2* expression in young EPCs was sufficient to suppress the survival, proliferation, migration, secretion and angiogenesis of these cells, while activating NRF2 in aged EPCs produced the opposite effects. Previous research has provided clues that NRF2 is associated with the decline in EPC function in diabetes, an age-related disease [17]. The current study directly identified NRF2 as a key determinant of EPC function during the sequence of ‘normal’ aging. We thus infer that NRF2 protects EPCs from aging and aged-related disease.

Increased ROS/reactive nitrogen species levels are hallmarks of aging and age-related diseases [20]. Puerarin has been reported to protect EPCs from the senescence induced by angiotensin II by activating the ERK1/2-NRF2 signaling pathway [21, 22]. We found that oxidative stress increased in EPCs during aging, while the activation of NRF2 reduced oxidative stress, as evidenced by ROS, malondialdehyde and SOD levels. We also observed aging-related changes in the senescence phenotype of EPCs, based on β-galactosidase, p16 and p21 levels. These data are in agreement with the studies mentioned above.

Classically, activated NRF2 induces multiple cell survival mechanisms, including antioxidant, anti-inflammatory and other cytoprotective pathways, by binding to antioxidant response elements in the promoter regions of its target genes [23, 24]. Lv et al. reported that xanthohumol, an NRF2-activating compound, effectively suppressed the LPS-activated TXNIP/NLRP3 inflammasome and the NF-κB signaling pathway in acute lung injury [25]. The NRF2/HO-1 pathway is known to reduce endoplasmic reticulum stress, inflammation and apoptosis in peripheral tissues [26]. Sulforaphane exerts its anti-inflammatory effects by activating the NRF2/HO-1 cascade in human THP-1 microglia-like cells [27]. However, other studies have suggested that NRF2 activation and HO-1 expression are both redox homeostatic factors and pro-inflammatory factors in NLRP3 inflammasome activation [28].

The present study had several limitations. Our experiments were conducted on a single animal strain, so additional species should be studied. In future experiments, EPCs isolated from human subjects could be used to increase the clinical relevance of the results. In addition, further studies are required to understand the mechanisms of NRF2 activity in EPCs, in view of the complexity of the potential signaling pathway.

In conclusion, we identified a notable reduction in EPC survival and regeneration during aging, and found that reduced NRF2 expression was a key contributor to this phenomenon. By determining the precise time period during which NRF2 expression is altered and impacts EPC function, we have discovered a fundamental aspect of EPC dynamics with aging. This study should lay the foundation for the development of targeted EPC-based strategies to promote healthy aging and treat age- related CVD.

## MATERIALS AND METHODS

### Animals

Mice were purchased from the Animal Experiment Center of Wuhan University, and were kept in the animal house of Tongji Medical College, Huazhong University of Science and Technology. All animals were handled according to the institutional animal care guidelines and the Guide for the Care and Use of Laboratory Animals published by Tongji Medical College. The studies were approved by the Institutional Animal Care and Use Committee of Tongji Medical College, Huazhong University of science and technology. When the mice had been raised to 3-4 months, 12–14 months or 21–24 months, the next animal experiment was carried out.

### EPC isolation and culture

The extraction of EPCs was based on a method described in the literature [29]. Briefly, the femurs and tibias of the mice were isolated and washed three times with sterile phosphate-buffered saline (PBS), and individual nuclear cells were washed out with a 1-mL syringe. The cells were cultured in EGM2 culture medium, and unattached cells were abandoned on the fourth day, while adherent cells continued to be cultured until the seventh day for the experiments.

### Characterization of EPCs

In accordance with previous publications [29, 30], adherent EPCs were characterized by dual staining for Dil-acLDL and lectin.

### Senescence-associated β-galactosidase assay

We assessed senescence by measuring β-galactosidase activity with a Senescence β-Galactosidase Staining kit (Beyotime Biotechnology, Shanghai, China) according to the manufacturer’s protocol. Briefly, treated cells were washed twice with PBS, fixed in fixation buffer for 15 min, washed three times with PBS and incubated with the freshly prepared staining mixture overnight at 37°C. Senescence-associated β-galactosidase-positive cells were detected under a bright field microscope (Nikon), and images were captured at 400× magnification. At least 200 cells were counted from different fields of the plate, and the percentage of senescence-associated β-galactosidase-positive cells was calculated. Each experiment was repeated three times.

### Surgical induction of unilateral hindlimb ischemia and blood flow analysis

Hindlimb ischemia was experimentally induced as described previously [31]. After being anesthetized with isoflurane, mice underwent left-hindlimb ischemic surgery. In this model, the femoral artery was surgically removed. We used laser speckle contrast analysis (PERIMED, PeriCam PSI, Sweden) to evaluate the blood flow recovery in the left and right legs and feet of the mice, before surgery and on postoperative days 0, 3, 7 and 14. The changes in the laser frequency and differently colored pixels were used to express the blood flow in each leg. The results of our quantitative analysis of leg blood flow are presented as the ratio of ischemic to non-ischemic laser speckle contrast analysis, in order to exclude data variations due to ambient temperature and light.

### Capillary density

On day 14 of the above experiment, capillary endothelial cells were identified with an anti-CD31 antibody (Abcam Laboratories, Cambridge, UK). The capillaries and muscle fibers in five random microscopic fields from three independent cross-sections of the adductor skeletal muscle in each animal were counted (n=5 per group), and the capillary density was expressed as the number of capillaries per muscle fiber [19].

### Assessment of EPC proliferation by EdU analysis

An EdU labeling/detection kit (Ribobio, Guangzhou, China) was used to evaluate the proliferation of EPCs according to the manufacturer’s protocol. Briefly, EPCs were grown in 24-well plates to about 5 × 10^4^ cells/well. After exposure to the described experimental conditions, EPCs were treated with a 50-μM EdU labeling medium and incubated for 4 h at 37°C under 5% CO_2_. The cells were then fixed with 4% paraformaldehyde, ruptured with 0.5% Triton X-100 and stained with an anti-EdU working solution. The nuclei were labeled with Hoechst 33258 (Guge, Wuhan, China), and fluorescent microscopy was performed (Olympus, Tokyo, Japan). The percentage of EdU-positive EPCs was calculated from five random fields-of-view per group.

### Matrigel tube formation assay

A tube formation assay was used to determine the angiogenic activity of bone marrow-derived EPCs, as described previously [32, 33], with minor modifications. Briefly, EPCs were plated at a density of 2 × 10^4^ cells/ well in 48-well plates precoated with 150 μL/well of Matrigel (BD Biosciences). After 15-18 h of incubation, tube formation was observed with a light microscope (Olympus, Tokyo, Japan). Images of tube morphology were taken in five random microscopic fields (100×), and the cumulative mean tube length per field-of-view (n=5) was quantified with ImageJ software (NIH).

### Measurement of NO and VEGF secretion

The secretory function of EPCs was evaluated based on NO and VEGF secretion, which were measured with an NO colorimetric assay kit (Enzo Life Sciences, Inc., USA) and a VEGF enzyme-linked immunosorbent assay kit (Bioswamp, Wuhan, China), respectively, according to the manufacturers’ instructions.

### Immunofluorescence assay

EPCs were plated on glass coverslips. After being washed with PBS, the cells were fixed with 4% paraformaldehyde, blocked with donkey serum, treated with an NRF2 antibody overnight, incubated with secondary antibodies (1:200) coupled to fluorochromes (Alexa 488; Antgene, Wuhan, China) and stained with Hoechst 33258 for the detection of nuclei. A confocal fluorescence microscope (Olympus, Tokyo, Japan) was used to observe the cells.

### Western blotting

Proteins were extracted by cell lysis and denatured for Western blotting. The divisible cell lysis products (50 μg) were separated on a 10% sodium dodecyl sulfate polyacrylamide gel, and were electro-transferred to a polyvinylidene fluoride membrane (Bio-Rad Laboratories, Inc.). The membrane was blocked with Tris-buffered saline-Tween buffer in 5% skimmed milk at room temperature for 1 h. Then, the membrane was incubated with the appropriate antibody (anti-NRF2 1:1000 or anti-beta-actin 1:2000, Cell Signaling Technology, USA; anti-p16, Arigo Biolaboratories Corp., China) at 4°C overnight. The membrane was washed with Tris-buffered saline-Tween buffer and incubated with a horseradish peroxidase-conjugated secondary antibody. After the membrane was extensively washed, a chemical luminescence detection system (ECL; Thermo Fisher Scientific, Inc., NY, USA) was used to quantify the protein levels.

### Real-time PCR

Real-time PCR was used for the quantitative analysis of mRNA levels. Total RNA was isolated from EPCs with Trizol reagent according to the manufacturer’s protocol (Life Technologies, Grand Island, NY, USA). Complementary DNA was synthesized with a PrimeScript™ RT reagent kit (Takara bio, Tokyo, Japan). A QuantiTect SYBR Green PCR kit (Qiagen, Redwood, USA) was used for quantitative real-time PCR on a Roche LightCycler ® 480 system (Roche Co., LTD., Switzerland).

### Measurement of ROS

Intracellular ROS levels were determined by imaging and flow cytometry analyses. For the imaging, following exposure to the described experimental conditions, EPCs were stained with 5 μmol/L CellROX^®^ Green Reagent (Thermo Fisher Scientific, Inc., Waltham, MA USA) and incubated for 40 min at 37°C. The medium was then removed, and the cells were washed three times with PBS. The cells were fixed with 4% formaldehyde for 15 min, and the nuclei were stained with Hoechst 33258. The cells were then examined with a fluorescent microscope (Olympus, Tokyo, Japan).

For the flow cytometry, EPCs were incubated with CellROX^®^ Green Reagent, washed with PBS three times and trypsinized. Flow cytometry was performed on a flow cytometer (FACSCalibur; BD Biosciences), and the data were analyzed with FlowJo software (FlowJo LLC, Ashland, OR, USA).

### SOD and malondialdehyde assays

The SOD activity and malondialdehyde content in the media were measured with commercially available kits and colorimetric assays (Nanjing Jiancheng Bioengineering Institute, Nanjing, China) according to the manufacturer’s protocols.

### Transfection with siRNA

After the optimal transfection conditions were determined (data not shown), siRNA (mouse Silencer Select siRNA for *Nrf2*, gene ID: 18024, Ribobio) was used to silence *Nrf2* expression, in accordance with the manufacturer’s protocol for the riboFECT™ CP Transfection Kit (Ribobio). The siRNA sequence (sense strand) used for *Nrf2* was CGACAGAAACCTCCATCTA. A Stealth RNAi Negative Control Duplex (Ribobio, Guangzhou, China) was used as a negative control. EPCs were collected 48 hours post-transfection for RNA isolation and 72 hours post-transfection for biological function measurements and protein isolation, respectively.

### Statistical analysis

Data are expressed as the mean ± standard error of the mean. Differences between groups were evaluated by either a two-tailed Student’s t test or one-way analysis of variance (ANOVA) followed by Dunnett’s T3 *post hoc* test. All statistical analyses were performed with SPSS software version 19.0 (IBM SPSS, Armonk, NY, USA). P<0.05 was considered to indicate a statistically significant difference.

## Supplementary Material

Supplementary Figure
