# Supplementary material for: Reduced NRF2 expression suppresses endothelial progenitor cell function and induces senescence during aging
Source: Aging (Albany NY). 2019 Sep 8;11(17):7021–35. doi: 10.18632/aging.102234 (PMC6756903; doi:10.18632/aging.102234)
Supplement: Supplementary Figure [file aging-11-102234-s001.pdf]

SUPPLEMENTARY FIGURE

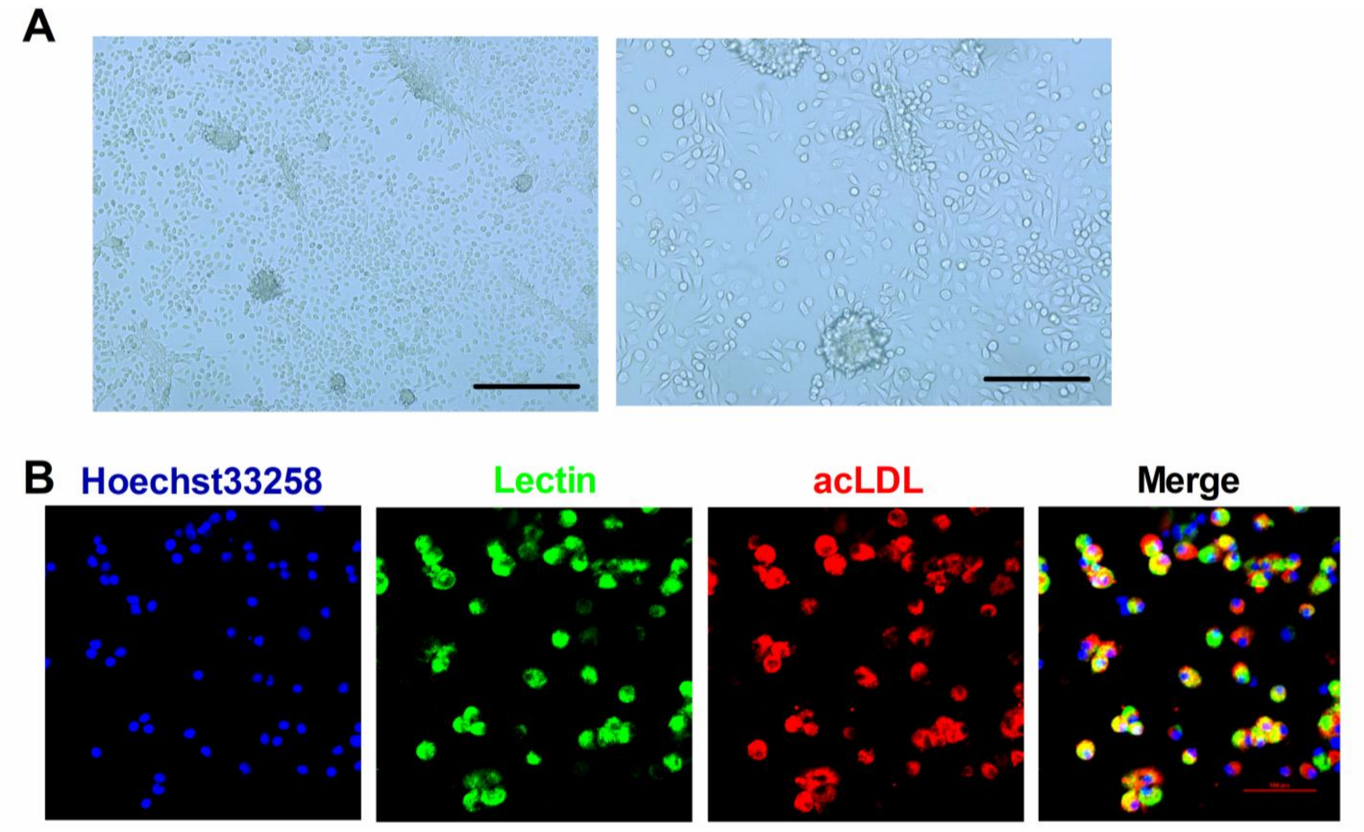

Supplementary Figure 1. Characterization of early EPCs. (**A**) The isolated mononuclear cells became spindle-shaped following 7 days of culture. Scale bar: 500 (left) and 200 (right) µm. (**B**) The EPCs were identified as double positive for Dil-acLDL (red) and lectin (green) following 7 days of culture. Scale bar: 100 µm.
